# Urban greenspace under a changing climate: Benefit or harm for allergies and respiratory health?

**DOI:** 10.1097/EE9.0000000000000372

**Published:** 2025-02-12

**Authors:** Tianyu Zhao, Joachim Heinrich, Michael Brauer, Nir Fulman, Nur Sabrina Idrose, Clemens Baumbach, Jeroen Buters, Iana Markevych, Beate Ritz, Rachel Tham, Bo-Yi Yang, Xiao-Wen Zeng, Samer Alashhab, Zhao-Huan Gui, Li-Zi Lin, Dennis Nowak, Maya Sadeh, Nitika Singh, Guang-Hui Dong, Elaine Fuertes

**Affiliations:** a Institute and Clinic for Occupational, Social and Environmental Medicine, University Hospital, LMU Munich, Munich, Germany; b Comprehensive Pneumology Center Munich (CPC-M), German Center for Lung Research (DZL), Munich, Germany; c Allergy and Lung Health Unit, Melbourne School of Population and Global Health, The University of Melbourne, Melbourne, Australia; d Institute for Health Metrics and Evaluation, University of Washington, Seattle, WA; e University of British Columbia, Vancouver, Canada; f Central Institute of Mental Health, Medical Faculty Mannheim, University of Heidelberg, Mannheim, Germany; g GIScience Research Group, Institute of Geography, Heidelberg University, Heidelberg, Germany; h Center of Allergy and Environment (ZAUM), Technical University of Munich, School of Medicine and Health & Helmholtz Munich, German Research Center for Environmental Health, Munich, Germany; i Institute of Psychology, Jagiellonian University, Krakow, Poland; j Research Group “Health and Quality of Life in a Green and Sustainable Environment,” Strategic Research and Innovation Program for the Development of MU—Plovdiv, Medical University of Plovdiv, Plovdiv, Bulgaria; k Environmental Health Division, Research Institute at Medical University of Plovdiv, Medical University of Plovdiv, Plovdiv, Bulgaria; l Department of Epidemiology, School of Public Health, University of California, Los Angeles, Los Angeles, California; m Department of Medicine, Melbourne Medical School, The University of Melbourne, Melbourne, Australia; n Joint International Research Laboratory of Environment and Health, Ministry of Education, Guangdong Provincial Engineering Technology Research Center of Environmental Pollution and Health Risk Assessment, Department of Occupational and Environmental Health, School of Public Health, Sun Yat-Sen University, Guangzhou, China; o Department of Epidemiology and Preventive Medicine, School of Public Health, Sackler Faculty of Medicine, Tel Aviv University, Tel Aviv, Israel; p The Taub Center for Social Policy Studies in Israel, Jerusalem, Israel; q Institute for Clinical Diabetology, German Diabetes Center (DDZ), Leibniz Center for Diabetes Research at Heinrich Heine University, Düsseldorf, Germany; r National Heart and Lung Institute, Imperial College London, London, United Kingdom; s MRC Centre for Environment and Health, Imperial College London, London, United Kingdom

**Keywords:** Greenspace, Climate change, Pollen, Volatile organic compounds, Ozone, Hypersensitivity, Lung function, Epidemiology

## Abstract

An increasing proportion of the world’s population lives in urban settings that have limited greenspace. Urbanization puts pressure on existing greenspace and reduces its access. Climate impacts, including increased temperature and extreme weather events, challenge the maintenance of urban vegetation, reducing its ecosystem services and benefits for human health. Although urban greenspace has been positively associated with numerous health indicators, the evidence for allergies and respiratory health is much less clear and mixed. To address these uncertainties, a workshop with 20 global participants was held in Munich, Germany, in May 2024, focusing on the impact of greenspace-related co-exposures on allergies and respiratory health. This narrative review captures key insights from the workshop, including the roles of urban greenspace in (1) climate change mitigation, (2) interactions with pollen, and (3) emissions of biogenic volatile organic compounds and their byproducts, such as ozone. Additionally, it presents research and stakeholder recommendations from the workshop. Future studies that integrate advanced greenspace exposure assessments and consider the interplay of greenspace with pollen and biogenic volatile organic compounds, along with their relevant byproducts are needed. Increased public awareness and policy actions will also be essential for developing urban greenspace that maximizes health benefits, minimizes risks, and ensures resilience amid a changing climate and rapid urbanization.

**What this study adds:**

This review enhances our understanding of the complex interactions among urban greenspace, climate change, allergies, and respiratory health. In particular, it discusses the role of greenspace in an urbanizing world facing ongoing climate change, with particular attention to the significant yet underexplored impacts of co-exposures such as pollen and biogenic volatile organic compounds, along with their byproducts like ozone. Key recommendations for research and stakeholders are also provided, which were developed from a workshop with global experts in the field.

## Introduction

Currently, 56% of the world’s population lives in urban settings, and this number is projected to increase to 70% by 2050.^1^ As urban areas expand to accommodate larger populations, the increased need for housing, transportation, and other infrastructure places a severe threat on access to urban greenspace (Box 1) in cities. For example, on a global scale, impervious surfaces within urban areas have increased from 24.3% to 25.9% (326,000 ha/year) over just 5 years (2012–2017). In contrast, the global average urban tree cover has decreased from 26.7% to 26.5% (around 40,000 ha/year).^2^

In addition to the pressures from population growth and expansion of the built environment, urban greenspace is further challenged by a changing climate. According to a study published in 2022,^3^ an estimated 56% and 65% of tree and shrub species, respectively, in 164 cities across 78 countries currently grow at suboptimal temperatures and precipitation conditions, and these pressures on vegetation are expected to increase under climate warming scenarios.

To maintain sufficient vegetation in urban areas, tree planting programs and other nature-based solutions, such as green roofs/walls, rain gardens, and constructed wetlands, have been implemented in cities worldwide^4,5^ and are at the forefront of many municipal climate mitigation actions.^6^ Urban greenspace, especially urban forests (i.e., forests or a collection of trees that grow within a city, town, or suburb),^7^ also have important roles in climate adaptation strategies, given their ability to mitigate urban heat islands (UHI).^8^

Despite these beneficial roles, urban greenspace (Box 1) can also have negative impacts, such as increasing exposure to pesticides and herbicides, serving as potential locations for crime, contributing to gentrification, harboring disease vectors, and releasing allergenic pollen.^9^ Indeed, although numerous studies support a beneficial relationship of urban greenspace on a broad range of human health indicators (Box 2),^10,11^ the evidence for allergies^12,13^ and respiratory health (Box 3),^14^ including lung function,^15,16^ is much less clear. These inconsistencies may be due to the largely unaddressed issues of plant species composition, as well as the often-neglected role of greenspace-related co-exposures, such as urban heat, pollen, and biogenic volatile organic compounds (BVOCs) plus their typical byproducts such as ozone. Thus, while it is possible that appropriate and equitable greening may benefit many health outcomes and minimize health inequalities,^17,18^ poorly planned greening efforts may exacerbate allergic and respiratory health problems, especially under climate threats such as extreme heat events.^19,20^

Box 1. **Urban greenspace**

Urban greenspace, including forests, meadows, residential yards, parks, grassy lawns, and green roofs, offers a wide range of ecosystem services to humans and the environment.^21,22^ They reduce urban heat, absorb greenhouse gases, mitigate pollution, and manage stormwater to prevent floods. They also support habitats for urban wildlife and contribute to biodiversity conservation.^23^ Urban greenspace also plays a significant role in promoting human health.^10,11^

Box 2. **Greenspace and health: epidemiological methodologies and pathways**

Numerous epidemiological studies support a beneficial relationship between urban greenspace and a broad range of human health indicators, from perceived health and quality of life to mental well-being, obesity, cardiovascular health, and mortality.^10,11^

Metrics used to evaluate greenspace exposure include subjective measures like self-reported exposure and visits to nature and objective measures such as the normalized difference vegetation index (NDVI), percentage of greenspace in a specific area, or proximity to the nearest greenspace.^10,11^

Three primary pathways have been proposed to explain the links between greenspace and improved health:^9^ (1) reducing harmful exposures, including decreasing air pollution, temperature, and noise levels; (2) restoring capacities, by reducing stress and improving cognitive attention through natural environments; and (3) building capacities, such as promoting physical activity and increasing microbial diversity.^24^

Box 3. **Allergies and respiratory health**

Allergies and respiratory diseases, such as asthma and chronic obstructive pulmonary disease, contribute to a high global disease burden. The World Health Organization estimates that 235 million people worldwide suffer from asthma, which is one of the leading causes of hospitalization among children.^25^ The Global Burden of Disease Study ranks chronic respiratory diseases among the top causes of disability-adjusted life years globally.^26^ The economic burden of these conditions is also substantial due to healthcare costs, lost productivity, and reduced quality of life.

Overall, the etiology of allergies and respiratory diseases is complex, including genetic predispositions and environmental factors, such as exposure to ambient air pollution and greenspace.^12–16^ To address this uncertainty, in May 2024, a workshop involving 20 participants from around the world—including Europe, Asia, the Middle East, North America, and Oceania—was held in Munich, Germany, with the aim of exchanging knowledge and ideas on the impact of greenspace-related co-exposures on associations between greenspace and health. The workshop focused on allergies, asthma, and lung function, given the highly heterogeneous literature regarding the impact of greenspace on these health outcomes.^12–16^ By critically examining the existing literature and identifying future research priorities, this workshop aimed to inform urban greening efforts so that greenspace can be designed to maximize population health benefits while minimizing negative impacts. Further details on the workshop and the methodology of this review can be found in the Supplemental Digital Content; http://links.lww.com/EE/A329.

**Figure 1.**
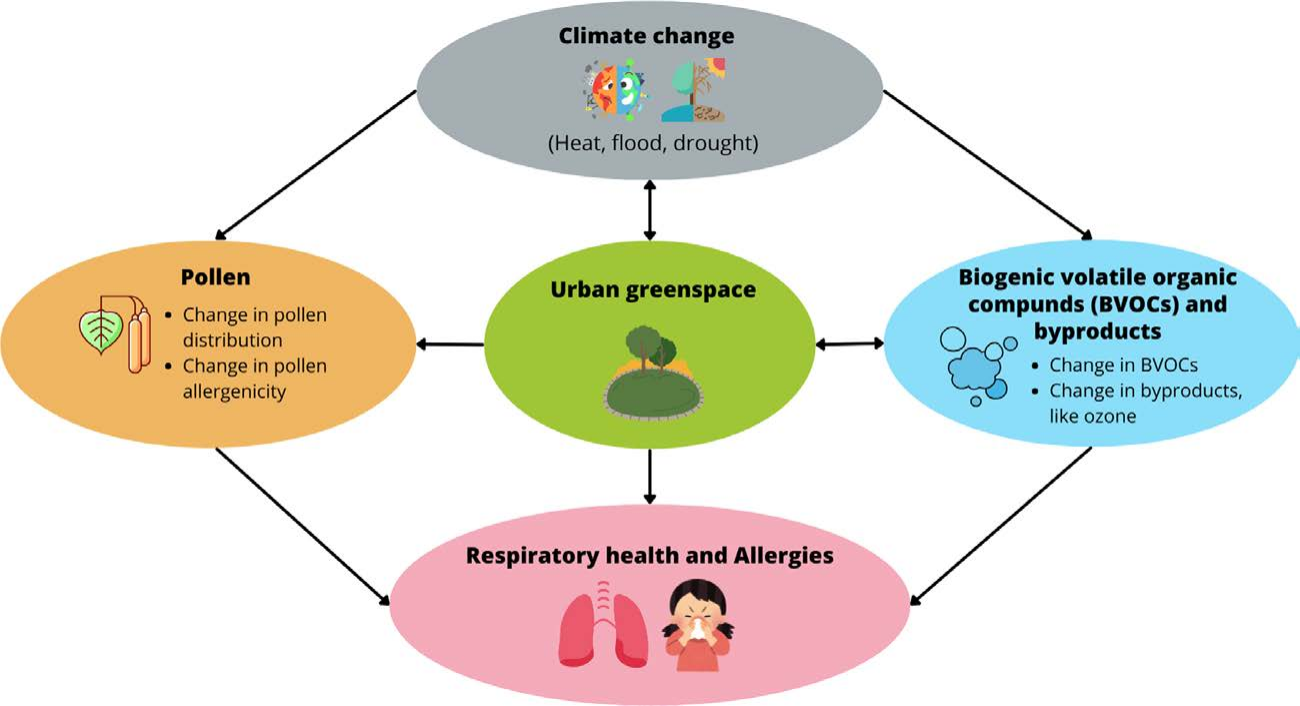
Pathways through which greenspace and its associated co-exposures may influence allergies and respiratory health in a changing climate.

This report summarizes the key points of this meeting and provides an in-depth discussion of the multifaceted relationships between greenspace, its related exposures, climate change, and allergies as well as respiratory health. Organized across five sections, this narrative review covers greenspace’s effects on climate change mitigation, interactions with pollen, and dynamics with BVOCs and one of their key byproducts, ozone. Additionally, this review outlines research priorities and stakeholder recommendations. The framework of this review is illustrated in Figure 1.

## Greenspace and a changing climate

### Greenspace as a mitigator of climate change

One of the primary ways that greenspace mitigates climate change impacts is by reducing the UHI effect.^27^ Urban areas with a dense concentration of buildings and impervious areas tend to be much warmer than surrounding rural areas with a similar climate, and the extreme UHI intensity is even 10 °C–15 °C higher.^28^ Urban greenspace helps counteract the UHI effect by providing shade and facilitating cooling through evapotranspiration, which can lower ambient temperature^29^ and reduce the risk of morbidity and mortality due to heat-related health issues.^27^ For example, a study across 93 European cities estimated that increasing urban tree coverage to 30% could reduce temperatures by 0.4 °C and prevent 2644 premature deaths.^27^ This cooling effect not only improves comfort and liveability outdoors in urban areas but also reduces the need for air conditioning, thereby lowering energy consumption and consequently greenhouse gas emissions.^30,31^ Interestingly, it appears that fragmented instead of aggregated greenspace layout may be more effective at reducing heatwaves in cities.^32^ However, researchers have also observed that wooded tree-covered areas can be warmer at night,^33,34^ and that a few types of greenspace, especially open grassland, may lead to colder temperatures in winter than what is observed in urban areas, although this effect appears to depend on latitude.^35^ Nonetheless, whether those inconsistent functions of greenspace are relevant for urban areas is unclear.

Urban greenspace also mitigates extreme weather events, such as flooding, and their health impacts. For instance, greenspace can absorb rainwater through permeable surfaces, reducing water runoff and decreasing flood risks.^36,37^ However, studies have also highlighted that urban greenspace in larger and separate areas may exacerbate flood risks.^36^ Depending on tree and shrub cover density and structure, greenspace can also act as natural windbreaks, lowering wind speeds and contributing to climate resilience in urban environments.^38^

These few aforementioned examples highlight the complex role of greenspace as a mitigator of climate change impacts and emphasize that various pathways are likely to be involved, depending on the greenspace characteristics (e.g., size, shape, and composition), geography, and other relevant factors (e.g., socioeconomic status of the residents in an urban area).

### Greenspace under climate change

Climate change poses significant challenges to health and the functionality of plants that make up greenspace in urban environments. Higher temperatures, altered precipitation patterns, and increased frequency of extreme weather events, including floods and droughts, can stress plant species, leading to reduced or stunted growth, increased vulnerability to pests and diseases, and higher plant mortality.^39,40^ Elevated carbon dioxide (CO_2_) levels can also make plants more appealing to certain sap-sucking pests, such as aphids, borers, and moths, by increasing the soluble carbohydrates in the plants.^41,42^ For instance, the emerald ash borer, an invasive beetle, has devastated ash trees across North America, leading to significant economic and ecological consequences. Donovan et al.^43^ highlighted how such tree loss can affect the urban population, including reduced air quality and increased heat stress, further exacerbating health issues. Moreover, increased temperatures or changing climatic conditions can accelerate the spread of pests and diseases that affect vegetation on a broad scale.^44,45^ As an example, *Biscogniauxia mediterranea* Kuntze, usually seen as harmless endophytic fungi, may cause charcoal disease on drought-stressed oaks (*Quercus* spp.).^46,47^ Overall, these changes compromise the ability of greenspace to provide cooling, air purification, and other ecosystem functions.

Climate change can also shift vegetation composition within urban greenspace, even resulting in a decline in its quality and diversity.^39^ By 2050, projections indicate that 2387 of 3129 assessed urban tree species will be at risk of poor growth and mortality due to shifts in mean annual temperatures, while 2220 species will suffer from changes in annual precipitation.^3^ On the other hand, while some plant species may become less viable under changing climate conditions, others that are more resilient to heat and drought may proliferate,^48–50^ especially drought-tolerant plants including olive (*Olea europaea* L.) and oak (*Quercus ilex* L.). Plant composition can be relevant to the production and emission of pollen^51^ and/or BVOCs.^52^ These two aspects are discussed in detail in the following subsections. Finally, changes in vegetation composition may also impact the esthetic and recreational value of greenspace,^53^ which are essential for mental health and cognitive function health benefits.^54,55^

## Health effects of greenspace with co-exposure to pollen

### Greenspace and pollen

As climate conditions change, the composition of vegetation in greenspace may shift,^39^ leading to influences on pollen distribution.^51,56–59^ While essential for plant reproduction, pollen can be a significant source of allergens as well as a carrier of microorganisms and pollutants.^60–62^ Allergenic plants, such as trees (birch, alder, hazel, oak, hornbeam, chestnut, and beech),^63^ grasses, and weeds, are commonly found in urban greenspace, making them an important source of allergenic pollen.^64^ Concerningly, ongoing climate change is changing the distribution, amount, timing, length, and severity of the pollen season, which will have important health impacts.^51,56–59^ Rising temperatures and increased levels of CO_2_ enhance plant growth and extend growing seasons.^65^ Climate change can also alter the timing of pollen seasons.^59,66,67^ Warmer temperatures are causing plants to start pollinating earlier in the year and can extend the duration of pollen seasons.^51,68^ These dynamics have already been observed in the pollen of trees, such as birch and oak, as well as grasses.^69–71^ Climate shifts will also expand the geographic range of allergenic plants.^71,72^ Ragweed, for example, is projected to spread into northern and eastern Europe under all climate scenarios, potentially increasing “high allergy risk” areas by 27%–100% by 2100.^73^

Furthermore, climate change may also affect pollen allergenicity. Air pollutants, such as ozone, can damage plant cells and promote the release of allergens from plants, such as pollen-associated lipid mediators, thereby enhancing their allergenicity.^74^ For example, a study of birch pollen collected from various locations in Munich found that the content of Bet v 1, the primary birch allergen, was positively correlated with ozone levels, and that extracts from these high-ozone areas triggered significantly larger wheal and flare reactions in skin prick tests compared with extracts from low-ozone areas.^75^ Elevated ambient CO_2_ levels were also shown to induce a stronger allergic response to ragweed.^76^

Finally, the increase in extreme weather events, particularly thunderstorms during pollen season, can provoke the sudden rupture of pollen into subpollen particles that are tiny enough to penetrate deeper into the airways, thereby increasing the intensity of asthma attacks in pollinosis patients, referred to as potentially fatal “thunderstorm asthma.”^77,78^

### Inconsistent health effects of greenspace with pollen

The health effects of greenspace as a potential source of pollen are substantial as 40% of the European population is sensitized to pollen allergens^60^ and thus at risk of hay fever (i.e., allergic rhinitis) symptoms and allergic asthma.^79,80^ However, most epidemiological studies investigating the effects of greenspace on allergies and respiratory health have not considered the types of plants in the greenspace and their allergenic potential, although pollen concentrations have been measured in some.^81,82^ Exceptions to this include Markevych et al.^83^ who linked childhood exposure to allergenic trees to an increased risk of allergic rhinitis in adulthood and a recent study, which reported that living close to birch trees, a common allergenic plant, is associated with poorer lung function in adults.^84^ These studies suggest that greenspace dominated by allergenic plant species poses respiratory health risks to individuals with pollen allergies, whereas those with a more diverse mix of species (i.e., fewer allergenic species in proportion) may provide a more balanced array of health benefits. Future studies, including pollen and/or allergenic tree exposure measures, are likely to shed light on the contradictory associations observed to date between greenspace and allergic and respiratory health outcomes.^83,85,86^ This approach is especially important when considering how climate change is likely to affect pollen-producing plants and pollen.

Moreover, while the triggering effects of pollen exposure on allergic symptoms and asthma are well established by numerous short-term effect studies,^79,80^ the adverse role of pollen exposure in causing long-term health effects is not well-known.^82,87,88^

## Health effects of greenspace with co-exposure to biogenic volatile organic compounds and ozone

### Greenspace, biogenic volatile organic compounds, and relevant byproducts

In addition to pollen, plants emit a wide range of BVOCs, which are primarily produced through metabolic processes occurring on the leaf surface. Isoprene is a BVOC predominantly emitted by broad-leaved deciduous trees, such as oak, poplar, and willow. Evergreen trees like pines, spruces, and firs release the BVOC monoterpenes, which can give these coniferous trees their characteristic pine scent.^89^ Similar to pollen, the production of BVOCs is being altered by a changing climate, given that BVOCs play crucial roles in biotic defence mechanisms against herbivores and pathogens, and for protection from abiotic stress such as heat, drought, extreme weather events, high atmospheric CO_2_, ozone, and enhanced ultraviolet radiation.^90^

Increasingly, studies have indicated that BVOCs are important precursors of secondary air pollutants.^91,92^ For instance, some tree species, such as oaks and eucalyptus, release BVOCs at high levels.^93^ In urban environments with high nitrogen oxides (NOx), these BVOC emissions can lead to increased ozone formation, potentially offsetting any air purification of anthropogenic ozone by greenspace.^94,95^ One research group reported that BVOCs, specifically biogenic terpenoids, account for approximately 60% of ozone and secondary organic aerosol (SOA) formation potential in Los Angeles during the summer, with this contribution expected to rise significantly as temperatures increase.^92^ A Chinese study found a slightly different result: isoprene made up the greatest contributions to ozone formation, while monoterpenes were responsible for the highest biogenic SOA production.^91^ The BVOCs-related secondary air pollutants are influenced not only by the relative abundance of biogenic and anthropogenic volatile organic compounds (VOCs) but also by the ratio between total VOCs and NOx.^91^

While trees emit BVOCs that are important ozone precursors, they can also directly absorb or react with ozone, lowering ambient ozone levels.^96,97^ This dual role of plants as both a sink and a source of ozone formation precursors is influenced by various factors, including the physiological status of the trees,^93^ which adds further complexity to the relationship between greenspace and ozone. Indeed, current studies have been unable to disentangle the complexities of what greenspace means for ozone levels, as these may depend on BVOC formation as well as the anthropogenic ozone absorption potential of the local vegetation.^52,93^

The structure of greenspace can also trap pollutants, including ozone, leading to higher localized concentrations.^98,99^ Dense canopies and restricted air circulation, particularly in areas like tree-lined busy roads,^100^ may exacerbate this trapping issue, potentially resulting in different health outcomes compared with more open or well-ventilated areas. In summary, beyond plant species, the structure of greenspace and its interactions with the surrounding biotope and environment significantly affect the functions of greenspace.

### Inconsistent health effects of greenspace with biogenic volatile organic compounds and ozone

Associations between BVOCs and potential human health effects are largely unknown and contrasting observations have been reported in experimental studies.^101,102^ Epidemiological evidence on BVOC-related health effects is thus far lacking, and it is unclear whether and to what extent BVOCs influence associations between greenspace and allergic or respiratory health effects.

While the byproducts of BVOCs, biogenic SOA and ozone, have been investigated,^93,103^ they received limited attention in epidemiological studies. However, given that exposure to high levels of ozone has been linked to various adverse health outcomes, including respiratory issues,^104,105^ allergies,^106,107^ cardiovascular problems,^108^ and mental health,^109^ it is plausible that greenspace-related ozone may confound associations between greenspace and allergic or other respiratory health effects. A deeper understanding of the interactions between greenspace, BVOC levels, and the resulting formation of ozone and other byproducts requires more detailed information about the greenspace composition (such as tree species and the physiological or health status of plants)^93^ as well as advanced modeling techniques to accurately assess BVOC exposures.

## Challenges and opportunities for research

### Advanced greenspace exposure assessment

Given the above complexities in estimating associations between greenspace and health under a changing climate, there is a clear need for more specific measurements of greenspace and its related co-exposures. Currently, the most used measures for greenspace are the NDVI and soil-adjusted vegetation index. While NDVI and similar metrics per se are not able to identify allergenic species, they can be utilized effectively by aligning them with pollen seasons and integrating local botanical surveys that can help map high-pollen-risk areas accurately.^110^ More recently, street view imagery has been used to develop the Green View Index, and satellite imagery acquired through the Google Earth Engine, for instance, may provide global spatial coverage, low data acquisition and processing costs, and longitudinal coverage over time.^111^

High-precision sensors, coupled with advancements in data science, facilitate the development of more granular spatial greenspace exposure metrics.^112–114^ Examples include the 1-m resolution RGB imagery (images in which each pixel is determined by the intensity of red, green, and blue colors) from Maxar WorldView and the 3-m resolution multispectral imagery from PlanetScope missions, which have been used to identify individual trees of allergenic species.^115^ Additionally, airborne sub-1-m and spaceborne 10-m resolution LiDAR (Light Detection and Ranging) sensors provide vertical measurements using information from aircrafts (like drones or plants) and satellites, respectively, and spaceborne radar sensors additionally offer cloud-penetrating capabilities. While accessing data from these sensors typically incurs costs and requires expertise from data scientists for processing, they enable detailed analysis and mapping of greenspace characteristics that are relevant to allergens and BVOCs. Moreover, multispectral satellite imagery and street view imagery have been used to classify crops.^116,117^ For instance, to monitor flowering periods on a large scale, recent studies have shown the effectiveness of using geotagged and dated photos from online platforms like Flickr.^118^

Large-scale datasets managed by specialized organizations facilitate cost-efficiency in data acquisition and processing as well as enhance research replicability. Examples of such datasets include the Meta Canopy Height Model at 1 m resolution,^119^ the US EnviroAtlas Meter-scale Urban Land Cover by the United States Environmental Protection Agency, which offers a 1-m resolution map distinguishing between trees, shrubs, and grass across 30 urban areas in the United States,^120^ and the UK Ordnance Survey Mastermap (https://www.ordnancesurvey.co.uk), which provides vegetation cover data at a 1–2-m resolution for deciduous and nondeciduous trees, scrub, and grassland throughout the United Kingdom. The newly available Europe pollen reanalysis data gives daily modeled pollen concentrations for all of Europe for several decades and is one example of a large-scale dataset on pollen.^121^

Finally, some larger cities, such as Munich, Vienna, Melbourne, and New York, monitor vegetation through manual measurement in public spaces,^83^ capturing data on tree counts, species identification, and vegetation health. However, field campaigns are costly, time-consuming, and typically exclude private green spaces.^114^ The potential to develop tree registries via voluntary reporting data on the OpenStreetMap platform is also promising.^122^

### Suggestions for future research

#### Studies on pollen and greenspace-related pollutants

Implementing advanced monitoring techniques to measure both pollen and plant emissions (e.g., BVOCs) at high temporal and spatial resolution in various types of greenspace will provide more accurate data that go beyond assessing only the presence and quantity of greenspace. As mentioned earlier, combining ground-based sensors with remote sensing technologies can help track spatial and temporal variations in pollen and other potential pollutants for use in epidemiological studies.

#### Multidisciplinary research

Multidisciplinary research that bridges environmental science, urban planning, geography, remote sensing, botany, ecology, and public health is crucial. Studies should consider not only the direct health impacts of greenspace but also the implications of types of plants, microclimates, geographic factors, and climate change on the function of the greenspace and its effects on human health.

#### Geographic bias

The current knowledge of the health effects of greenspace is mainly based on studies from Europe, North America, Australia, and more recently also from China and other parts of Asia. However, there are limited studies from the African and South American continents published to date,^10,11^ which confines the generalizability of current knowledge. Notably, our workshop also lacked participants from these regions. In general, low- and middle-income countries are undergoing massive urbanization, and how urban greenspace is shaped in these areas has the potential to influence health to a large degree and can, for example, work toward reaching the sustainable development goals established by the United Nations.^123,124^

#### Potential “negative side effects” of greenspace

As aforementioned, greenspace might be associated with adverse impacts, such as the spread of disease vectors, the use of pesticides and herbicides, and places for crime.^9^ These “negative side effects” of greenspace could explain some of the inconsistent findings in epidemiological studies and require further attention.

### Suggestions for stakeholders

#### Health education and health promotion

Increasing public awareness about the effects and interactions of climate change, pollen, and BVOCs and their byproducts, like ozone, on greenspace and health can empower individuals to take proactive measures. As an example, health professionals can provide guidance on managing allergies and asthma before and during peak pollen seasons.

#### Monitoring and forecasting

Climate change models and environmental monitoring systems can provide valuable information about expected temperature increases, precipitation changes, and extreme weather events.^125^ Enhanced monitoring and forecasting of climate conditions and their impacts on greenspace and pollen can guide the management and design of greenspace and help mitigate the health effects of climate change.

#### Policy and urban planning implications

Policymakers and urban planners can build on existing knowledge of greenspace, pollen, BVOCs, and their byproducts to design and manage urban greenspace that optimize health benefits while minimizing potential risks. Meanwhile, these approaches should also consider species diversity and economic considerations.^98^ Box 4 summarizes some of the main points as examples.

Box 4. **Recommendations for urban planning**

(1) Select appropriate plants:^93,98,126^ prioritizing vegetation with lower levels of allergenic pollen in sensitive areas, such as near the homes or schools of vulnerable children, might be a relevant approach.^127^ Specific recommendations regarding the selection of particular plants are further complicated by the complex interactions between plant species, pollutant types, site-specific environmental conditions, and climate change. Nonetheless, it remains essential to strike a balance between minimizing potential health risks and promoting biodiversity when choosing appropriate vegetation.
(2) Optimize greenspace layout:^128,129^ designing greenspace to enhance airflow and reduce pollutant trapping while ensuring accessibility and safety.
(3) Integrate green infrastructure:^27,94^ combining greenspace with other forms of green infrastructure, such as vertical greening systems,^130^ to create a synergistic effect on air quality and urban microclimates. This includes designing greenspace that considers plant species, structure, and morphology of the greenspace, as well as climate scenarios, for example, increased temperatures and altered precipitation patterns.
(4) Ensure greenspace accessibility:^131,132^ designing greenspace and green infrastructure in urban areas to ensure that those of lower socioeconomic status and/or more vulnerable to the impacts of climate change also reap its benefits,^133^ while avoiding green gentrification.^134^

## Conclusions

Greenspace is essential for urban environments and human health. However, the allergic and respiratory health effects of greenspace are likely to depend on its characteristics, which have been largely ignored to date, and will be influenced by climate change as well as the direct and interactive effects of pollen, BVOCs, and relevant byproducts, including ozone. Comprehensive research and multidisciplinary approaches are needed to better understand these relationships, with a focus on utilizing advanced greenspace exposure assessments and considering the interplay of these various environmental factors under climate change scenarios. Increased public awareness and policy interventions are necessary to design and manage urban greenspace that maximizes health benefits, mitigates risks, and ensures resilience in the face of a continuously changing climate and rapid urbanization.

## Conflicts of interest statement

The authors declare that they have no conflicts of interest with regard to the content of this report.

## Supplementary Material


