# Supplementary material for: Urban greenspace under a changing climate: Benefit or harm for allergies and respiratory health?
Source: Environ Epidemiol. 2025 Feb 12;9(2):e372. doi: 10.1097/EE9.0000000000000372 (PMC11826049; doi:10.1097/EE9.0000000000000372)
Supplement: Supplementary file 1 [file ee9-9-e372-s001.pdf]

Supplemental Digital Content

Urban greenspace under a changing climate: benefit or harm for allergies and respiratory health?

Tianyu Zhao [a,b,*], Joachim Heinrich [a,b,c], Michael Brauer [d,e], Nir Fulman [f, g], Nur Sabrina Idrose [c], Clemens Baumbach [a,b], Jeroen Buters [h], Iana Markevych [i, j, k], Beate Ritz [l], Rachel Tham [c, m], Bo-Yi Yang [n], Xiao-Wen Zeng [n], Samer Alashhab [h], Zhao-Huan Gui [n], Li-Zi Lin [n], Dennis Nowak [a,b], Maya Sadeh [o, p], Nitika Singh [q], Guang-Hui Dong [n], Elaine Fuertes [r, s]

a. Institute and Clinic for Occupational, Social and Environmental Medicine, University Hospital, LMU Munich, Munich, Germany
b. Comprehensive Pneumology Center Munich (CPC-M), German Center for Lung Research (DZL), Munich, Germany
c. Allergy and Lung Health Unit, Melbourne School of Population and Global Health, The University of Melbourne, Melbourne, Australia
d. Institute for Health Metrics and Evaluation; University of Washington, Seattle
e. University of British Columbia; Vancouver, Canada
f. Central Institute of Mental Health, Medical Faculty Mannheim, University of Heidelberg, Mannheim, Germany
g. GIScience Research Group, Institute of Geography, Heidelberg University, Heidelberg, Germany
h. Center of Allergy and Environment (ZAUM), Technical University of Munich, School of Medicine and Health & Helmholtz Munich, German Research Center for Environmental Health, Member of the German Center of Lung Research (DZL), Member of the Immunology and Inflammation Initiative of the Helmholtz Association, Munich, Germany
i. Institute of Psychology, Jagiellonian University, Krakow, Poland

j. Research Group "Health and Quality of Life in a Green and Sustainable Environment", Strategic Research and Innovation Program for the Development of MU - Plovdiv, Medical University of Plovdiv, Plovdiv, Bulgaria
k. Environmental Health Division, Research Institute at Medical University of Plovdiv, Medical University of Plovdiv, Plovdiv, Bulgaria
l. Department of Epidemiology, School of Public Health, University of California, Los Angeles, Los Angeles, CA
m. Department of Medicine, Melbourne Medical School, The University of Melbourne
n. Joint International Research Laboratory of Environment and Health, Ministry of Education, Guangdong Provincial Engineering Technology Research Center of Environmental Pollution and Health Risk Assessment, Department of Occupational and Environmental Health, School of Public Health, Sun Yat-Sen University, Guangzhou 510080, China
o. Department of Epidemiology and Preventive Medicine, School of Public Health, Sackler Faculty of Medicine, Tel Aviv University, Tel Aviv, Israel
p. The Taub Center for Social Policy Studies in Israel, Jerusalem, Israel
q. Institute for Clinical Diabetology, German Diabetes Center (DDZ), Leibniz Center for Diabetes Research at Heinrich Heine University, Düsseldorf, Germany
r. National Heart and Lung Institute, Imperial College London, London, United Kingdom
s. MRC Centre for Environment and Health, Imperial College London, London, United Kingdom

* Corresponding author:

Dr. Tianyu Zhao

Institute and Clinic for Occupational, Social and Environmental Medicine

University Hospital LMU Munich

Ziemssenstraße 5, 80336 Munich

Germany

Email address: Tianyu.Zhao@med.uni-muenchen.de; zhaoty2009@yahoo.com

Contents

Methods 4
- 1. Criteria for participant selection 4
- 2. Workshop structure 4
- 3. Development of priority research areas and recommendations 5
- 4. Literature review process 5

Workshop Agenda 7

Methods

This section provides a description of the methodology used in generating this narrative review. While systematic reviews follow predefined guidelines and criteria, this manuscript employed a non-systematic approach to synthesize evidence, given the broad and evolving nature of the topic. The objective of this review is to present a broad discussion of previous research, highlight current knowledge gaps, and offer suggestions for future work and policymaking.

1. Criteria for participant selection

Participants for the workshop were selected based on their expertise in the fields of greenspace, respiratory health, and allergic diseases. The selection process aimed to ensure a broad representation of geographic regions and research disciplines. Invitations were extended to researchers with significant experience and publications in these areas, as well as those who had been involved in relevant research projects.
Ultimately, 20 participants from various regions, including Europe, Asia, the Middle East, North America, and Australia, attended the workshop. Unfortunately, participants from Africa and South America were not represented. This limitation is acknowledged in the manuscript (section *Geographic bias*).

More details on the workshop participants, such as their work locations and presentation topics, can be found in the *Workshop Agenda* included below.

2. Workshop structure

The workshop was held over two days in May 2024 in Munich, Germany. It included presentations by participants on key topics, followed by moderated discussions aimed at identifying research gaps and formulating recommendations. Rapporteurs documented the discussions, which formed the basis for the narrative review.

There were four key themes, and several presentations were given per theme (see *Workshop Agenda*):

(1) Current knowledge on greenspace and health in the light of climate change

(2) Greenspace and ozone

(3) Greenspace and pollen

(4) Novel greenspace-related metrics

3. Development of priority research areas and recommendations

The recommendations and priority research areas presented in the manuscript were developed using a collaborative process involving all workshop participants. During the workshop, participants discussed and proposed potential future research directions. These were recorded and summarized by the workshop Rapporteurs and circulated to all participants for review. Using this information, a core writing team (TZ, JH, and EF) led the drafting of the manuscript. All participants had the opportunity to contribute, review, and comment on three draft versions of the manuscript to ensure their perspectives were accurately reflected. Any disagreements were resolved through iterative discussions and consensus-building among all co-authors.

The specific roles and contributions of the authors are detailed in the *Credit Authorship Contribution Statement* section of the manuscript.

4. Literature review process

Although this manuscript is not a systematic review, existing literature was reviewed to provide context, support workshop discussions, and help identify key knowledge gaps.

The general literature review process involved:

Databases	PubMed, Web of Science, and Google Scholar.
Timeframes	Papers published up to May 2024 were considered.
Keywords	Searches included terms such as “greenspace,” “climate change,” “respiratory health,” “allergic diseases,” “air pollution,” “Pollen,” and "volatile organic compounds."
Inclusion/Exclusion Criteria	Papers were included if they were peer-reviewed and relevant to the themes discussed in the workshop and the manuscript. Non-peer-reviewed sources, such as conference abstracts and reports, were excluded.

Workshop Agenda

The workshop agenda is provided below. It has been modified slightly from its original form for publication purposes. However, the core content remains unchanged.

Greenspace and related exposures (ozone, pollen, and temperature) for health: Expert workshop

**Workshop organizers:**	Institute and Outpatient Clinic for Occupational, Social and Environmental Medicine, Ludwig Maximilian University of Munich (Joachim Heinrich, Tianyu Zhao)
**Participants**	Ludwig Maximilian University:<br>Joachim Heinrich, Dennis Nowak, Tianyu Zhao<br>Sun Yat-sen University:<br>Guang-Hui Dong, Bo-Yi Yang, Xiao-Wen Zeng, Zhao-Huan Gui, and Li-Zi Lin<br><br>Invited experts:<br>Mr. Clemens Baumbach (clemens.baumbach@gmx.net), *Jagiellonian University, Poland*<br>Prof. Michael Brauer, michael.brauer@ubc.ca, *University of British Columbia, Canada*<br>Prof. Jeroen Buters, buters@tum.de, *Technical University Munich, Germany*<br>Dr. Elaine Fuertes, e.fuertes@imperial.ac.uk, *Imperial College London, United Kingdom*<br>Dr. Nir Fulman, nir.fulman@uni-heidelberg.de, *University of Heidelberg, Germany*<br>Dr. Nur Sabrina Idrose, Sabrina.idrose@unimelb.edu.au, *University of Melbourne, Australia*<br>Prof. Iana Markevych, iana.markevych@uj.edu.pl, *Jagiellonian University, Poland*<br>Prof. Beate Ritz, britz@ucla.edu, *University of California, Los Angeles, United States*<br>Ms. Maya Sadeh, mayas@taubcenter.org.il, *Tel-Aviv University, Israel*<br>Dr. Rachel Tham, rachel.tham@unimelb.edu.au, *University of Melbourne, Australia*
**Contact:**	Tianyu Zhao, Tianyu.Zhao@med.uni-muenchen.de
**Background and general goal of the workshop:**	There are two mega trends worldwide for future decades: Increasing urbanization with decreasing presence of greenspace in metropolitan areas and climate change

	Recent studies report associations generally supporting a beneficial relationship of greenspace (i.e., vegetation intensity and/or green space) on a broad range of health indicators, including perceived health and quality of life, mental health, birth weight, respiratory health, obesity, and cardiovascular health. However, the results of epidemiological studies on greenspace and lung function and, in addition, allergies are mixed. The reasons for this are unclear so far. Greenspace-related co-exposures, e.g., ozone and pollen, and/or other regional characteristics have the potential to clarify the reasons for the mixed findings.<br><br>To date, no specific forum has taken place to allow the exchange of experience and ideas on these greenspace-related co-exposures in different regions of the world (with a focus on Germany and China). We thus propose to host a workshop focusing on current knowledge on the association between greenspace and the role of co-exposures such as ozone, pollen, and temperature for selected health indicators for respiratory health and specifically for lung function and allergies and eventually for cardiorespiratory health
**The specific objectives:**	1. Elucidating the role of co-factors like ozone, temperature,-and pollen, and temperature exposure for respiratory and cardiovascular health.<br>2. Identifying knowledge gaps and developing innovative research plans for future initiatives and collaborations that will explore the role of concomitant exposure for the development of respiratory and cardiovascular diseases.
**Expected outcomes:**	1. Publication of a workshop report in a peer-reviewed journal<br>2. Education and training for post-docs and doctoral students by dissemination of workshop results
**Format of workshop**	Four sections with presentations and discussion within two days<br>Presentation: 20 min (15 min presentation +5 min Q&A)<br>Discussion: Chair-leading long discussions with specific discussion points assigned to experts and free discussion of all participants.<br><br>Four assigned rapporteurs shall summarize the section and, accordingly, draft summaries that can be used in the workshop report.

WORKSHOP STRUCTURE

Day 1: a. Current knowledge on greenspace and health in the light of climate change (09:00 – 12:30)

<table>
<tr><th colspan="2">Welcome session and Welcome coffee</th></tr>
<tr><td>09:00 – 09:05</td><td>Welcome
Dennis Nowak</td></tr>
<tr><td>09:05 – 09:10</td><td>Welcome
Guang-Hui Dong</td></tr>
<tr><td>09:10 – 09:15</td><td>Introduction of workshop aim
Joachim Heinrich</td></tr>
<tr><th colspan="2">Morning session, Chair *Beate Ritz*</th></tr>
<tr><td>09:15 – 09:35</td><td>Current knowledge on greenspace and health: an overview
Bo-Yi Yang</td></tr>
<tr><td>09:35 – 09:55</td><td>Current knowledge on greenspace, temperature, climate change, and health: an overview
Li-Zi Lin</td></tr>
<tr><td>09:55 – 10:05</td><td>Break</td></tr>
<tr><td>10:05 – 10:25</td><td>Urban greenspace and population health: looking to the future
Michael Brauer</td></tr>
<tr><td>10:25 – 10:45</td><td>Greenspace and potential mechanisms (including lung and allergies)
Maya Sadeh</td></tr>
<tr><td>10:45 – 11:00</td><td>Break</td></tr>
<tr><td>11:00 – 12:20</td><td>*Chair Beate Ritz*
Moderated panel discussion on climate change and expected health hazards:
• Potential explanations regarding unexpected results of exposure to greenspace health outcomes
• urban greenspace and climate change
• Different types of interactions with the physical environment by gender and SES and their influence on health outcomes
• Exploring physical manifestation of psychosocial pathways in which greenness may affect health
• How much green is needed for health, discussion of arid vs temperate climates
• Can current epidemiological studies explore health effects of climate change?</td></tr>
<tr><td>12:20 – 12:30</td><td>Rapporteur: *Bo-Yi Yang*</td></tr>
<tr><td>12:30 – 14:00</td><td>Lunch break</td></tr>
</table>

Day 1: b. Greenspace and ozone (14:00 – 17:30)

Afternoon session, Chair *Rachel Tham*	
14:00 – 14:20	Ozone and climate change. The perspective of plant ecology<br>*Xiao-Wen Zeng*
14:20 – 14:40	Greenspace, ozone, and allergies<br>*Zhao-Huan Gui*
14:40 – 14:55	Break
14:55 – 15:15	Greenspace, ozone, and lung function<br>*Tianyu Zhao*
15:15 – 15:35	Greenspace, ozone, treepollen, and lung function in Germany<br>*Clemens Baumbach*
15:35 – 15:50	Coffee Break
15:50 – 17:20	*Chair Rachel Tham*<br>Moderated panel discussion on greenspace and ozone:<br>• Does more inner-city green increase the concentration of ozone in the inner cities and or VOC?<br>• Temperature/climate change and ozone<br>• Types of greenspace, ozone, and health effects<br>• Electric vehicles and ozone in (especially southern Chinese) inner cities<br>• Does treepollen exposure explain the adverse effects of living in a green neighborhood on lung function decline and prevalence of allergic symptoms?
17:20 – 17:30	Rapporteur: *Xiao-Wen Zeng*

Day 2: a. Greenspace and pollen (09:00 – 12:30)

Morning session, Chair *Jeroen Buters*	
09:00 – 09:20	Pollen and climate change (and greenspace)<br>*Jeroen Buters*
09:20 – 09:40	Greenspace, aeroallergens, ozone and allergic respiratory health in the Asia-Western Pacific region<br>*Rachel Tham*
09:40 – 10:00	Pollen, greenspace, and asthma in the UK Biobank<br>*Elaine Fuertes*
10:00 – 10:10	Break
10:10 – 10:30<br>20 min	Greenspace, pollen, and cardiorespiratory health<br>*Maya Sadeh*
10:30 – 10:50	Greenspace, aeroallergens, ozone and cardiovascular health in the Asia Western-Pacific region<br>*Nur Sabrina Idrose*
10:50 – 11:00	Coffee Break
11:00 – 12:20	*Chair: Jeroen Buters*<br>Moderated panel discussion on the role of pollen:<br>• Does treepollen exposure explain the adverse effects of living in a green neighborhood on lung function decline and prevalence of allergic symptoms?<br>• What are the most likely pathways, and are they the same/similar for all outcomes?<br>• Can we design studies to improve our understanding of the potential mechanisms (biomarkers, experimental studies, innovative epidemiological studies)?<br>• How can we best approach the potential interacting or confounding effects of climate, air pollution, and greenness on health?<br>• Can we design studies to improve our understanding of the potential mechanisms (biomarkers, experimental studies, innovative epidemiological studies)?<br>• How can we best approach the potential interacting or confounding effects of climate, air pollution, and greenness on health?
12:20 – 12:30	Rapporteur: *Clemens Baumbach*
12:30 – 14:00	Lunch break

Day 2: b. Novel greenspace-related metrics (14:00 –17:30)

Afternoon session, Chair *Michael Brauer*	
14:00 – 14:20	Residing near allergenic trees can increase risk of allergies later in life<br>*Iana Markevych*
14:20 – 14:40	Greenspace, ozone, BVOC, and health in China<br>*Bo-Yi Yang*
14:40 – 14:50	Break
14:50 – 15:10	Advances in Green space exposure assessment using street view images<br>*Nir Fulman*
15:10 – 15:30	Indoor plants and mental health<br>*Tianyu Zhao*
15:30 – 15:40	Rapporteur: *Nir Fulman*
15:40 – 15:50	Coffee Break
15:50 – 17:20	Wrap up<br>*Chair: Michael Brauer*
	Summary reports (written, if possible) of the 4 rapporteurs<br>*Bo-Yi Yang, Xiao-Wen Zeng, Clemens Baumbach, Nir Fulman*<br><br>And general discussion of suggestions for the workshop publication
17:20 – 17:30	Workshop Rapporteur and concluding remarks<br>*Tianyu Zhao*
